# Local production of pharmaceuticals in Africa and access to essential medicines: 'urban bias’ in access to imported medicines in Tanzania and its policy implications

**DOI:** 10.1186/1744-8603-10-12

**Published:** 2014-03-10

**Authors:** Phares GM Mujinja, Maureen Mackintosh, Mary Justin-Temu, Marc Wuyts

**Affiliations:** 1 Muhimbili University of Health and Allied Sciences, PO Box 65105, Dar es Salaam, Tanzania; 2 Department of Economics, Faculty of Social Sciences, The Open University, Walton Hall, Milton Keynes MK7 6AA, UK; 3 International Institute of Social Studies, Erasmus University Rotterdam, P.O. Box 29776, 2502 LT The Hague, The Netherlands; 4 REPOA, 157 Mgombani Street, Regent Estate, P.O. Box 33223, Dar es Salaam, Tanzania

**Keywords:** Local production of medicines, Access to medicines, Africa, Tanzania, Urban bias

## Abstract

**Background:**

International policy towards access to essential medicines in Africa has focused until recently on international procurement of large volumes of medicines, mainly from Indian manufacturers, and their import and distribution. This emphasis is now being challenged by renewed policy interest in the potential benefits of local pharmaceutical production and supply. However, there is a shortage of evidence on the role of locally produced medicines in African markets, and on potential benefits of local production for access to medicines. This article contributes to filling that gap.

**Methods:**

This article uses WHO/HAI data from Tanzania for 2006 and 2009 on prices and sources of a set of tracer essential medicines. It employs innovative graphical methods of analysis alongside conventional statistical testing.

**Results:**

Medicines produced in Tanzania were equally likely to be found in rural and in urban areas. Imported medicines, especially those imported from countries other than Kenya (mainly from India) displayed 'urban bias’: that is, they were significantly more likely to be available in urban than in rural areas. This finding holds across the range of sample medicines studied, and cannot be explained by price differences alone. While different private distribution networks for essential medicines may provide part of the explanation, this cannot explain why the urban bias in availability of imported medicines is also found in the public sector.

**Conclusions:**

The findings suggest that enhanced local production may improve rural access to medicines. The potential benefits of local production and scope for their improvement are an important field for further research, and indicate a key policy area in which economic development and health care objectives may reinforce each other.

## Introduction

This article contributes new analysis and findings to the debate concerning the scope for local pharmaceutical manufacturing in Africa to provide benefits to the population in terms of improved access to medicines. Using existing data sets, it presents empirical evidence of 'urban bias’ in the distribution of imported essential medicines in Tanzania : while medicines manufactured in Tanzania are equally likely to be found in rural and urban outlets, imported medicines – especially those manufactured outside the region – are less likely to be available in rural areas. Since rural areas contain a disproportionate share of extreme poverty in Tanzania, these are findings of real concern for health policy. The article explores possible explanations of these observations, and identifies policy implications concerning the potential benefits of linking of health and industrial policy by African policy makers, and potential health benefits were donors to pay greater attention to sourcing from local producers.

### Background

Over the last decade, there has been a huge increase in international funding for access to essential medicines, with a particular focus on HIV, TB and malaria in Sub-Saharan Africa [1-4]. Large scale international procurement of medicines is sourced predominantly from Indian manufacturers [5-7], a procurement strategy justified by international policy makers on grounds of lower prices and improved quality control. Many international analysts remain critical of the scope for locally produced medicines to improve access to medicines in African contexts [8-10].

However, there is increasing policy interest, among African and international policy makers, researchers, and some donors and suppliers, in the scope for improved local African pharmaceutical supply and its potential benefits [11]. The World Health Organization (WHO) is leading a project on the topic funded by the European Union in collaboration with UNCTAD [12-14]. There is currently substantial international support from UNIDO and from some aid agencies, notably GIZ, for strengthening local pharmaceutical production in Africa [15-17]. The African Union has been leading on the development of a Pharmaceutical Plan for Africa, supported by UNIDO and working with NEPAD and COHRED [18,19]. There is active policy development by a number of African governments, including Ghana, Uganda and South Africa, and involving pharmaceutical manufacturers’ associations such as SAGMA [16,20,21]. Donors and international NGOs, including PEPFAR and Action Medeor, have been developing local pharmaceutical procurement in a number of Africa countries^a^. There remains however limited evidence to date of the nature and extent of benefits of local production in terms of medicines access [10].

## Methods and theory

### Methods

The main sources of the findings presented in this article are the WHO/HAI medicines surveys conducted each year in Tanzania during 2006-2009^b^. The data used here are from the 2006 and 2009 surveys. The data collection, following WHO/HAI guidelines for the surveys at that time [22] included both price data and data on manufacturer and country of origin of each of 40 tracer medicines covered in both the 2006 and 2009 surveys^c^. A total of 96 facilities in public and faith-based sectors and private shops, in four regions of Tanzania, were included in both surveys^d^. Sample sizes used to calculate probabilities in this article are: for 2006, rural, 40 medicines × 47 outlets = 1880 observations; urban, 40 medicines × 46 outlets = 1840 observations; for 2009, rural, 40 medicines × 47 outlets = 1880 observations; urban: 40 medicines × 45 outlets = 1800 observations. The manufacturer and country of origin data from the two surveys were coded and analysed, and the article also uses the price data in the findings and discussion. Prices per dose were calculated using Tanzanian standard treatment guidelines for dosages [23].

In addition, this article also draws on field data collected during 2006-7, as part of a research project on the role of non-governmental public action in medicines access^e^, and on interviewing for an unpublished report to UNITAID^f^.

The data were analysed using Stata for quantitative analysis and NVivo for qualitative data. A specific feature of this article is that it combines conventional statistical two-sample tests for proportions with the use of innovative graphical methods to display and explore the extent of urban bias over the range of medicines. These graphical methods avoid the loss of information resulting from averaging data across medicines, displaying results for each medicine in a parsimonious manner. The basic rationale for adopting this method is that 'there is no single statistical tool that is as powerful as a well-chosen graph’ [24]. More specifically, the curvatures of patterns (including the location of unusual data points) that can easily be detected in a visual display are often rendered obscure or less easy to judge from an equivalent table of the data [24] p.1. This is particularly the case when the analysis requires looking at patterns across a range of 40 medicines.

### Theory

The analytical framework for the discussion of our results in this paper draws both on economic analysis of health and medicines markets, and on the 'urban bias’ literature in development studies. The concept of 'urban bias’ [25] has a contested but still productive role in development theory and policy [26-28]. The concept characterises processes that are biased against rural areas in a manner that undermines both economic efficiency and equity. We are concerned in this article not with price 'twists’ [25], nor centrally with 'public-expenditure urban bias’ [27] p.229 though the latter may be part of the policy story, but rather we draw on the more general concept of 'distributional urban bias’ [26]. This descriptive use of 'urban bias’ is found in some of the health and development literature with reference to inequity in the distribution of public provision and NGO activity in health^g^. We analyse here the extent to which distribution mechanisms for imported medicines – but not for locally produced medicines – appear systematically to disfavour the rural population.

Since the rural areas in Tanzania include the majority of the households in severe poverty in the country [29], urban bias in essential medicines availability is of policy concern. Recognising the high levels of commercialisation and charging within the Tanzanian health system [30-34], we explore in this article the extent to which our findings on urban bias in imported medicines distribution may be explained by lesser purchasing power in rural areas. We also use qualitative evidence and evidence from other studies to consider the role of consumer and clinician preferences in the outcomes, as well as the organisation of public and private wholesaling.

## Results

This section presents systematically the findings from the WHO/HAI survey data for 2006. Given the innovative graphical method of analysis, we do not present in the text the comparable graphs for 2009, in order to prevent 'graph overload’ in the exposition. We comment on and assess at each stage in the text the similarities and differences between the 2006 and 2009 survey findings, and we present the statistical test results for the two years, to confirm that the 2006 findings are not unique to that year. We also make available the 2009 comparative figures in a web-based appendix [Additional file 1] for readers interested in confirmation and further detail.

### Urban bias in access to essential medicines

**Figure 1:**
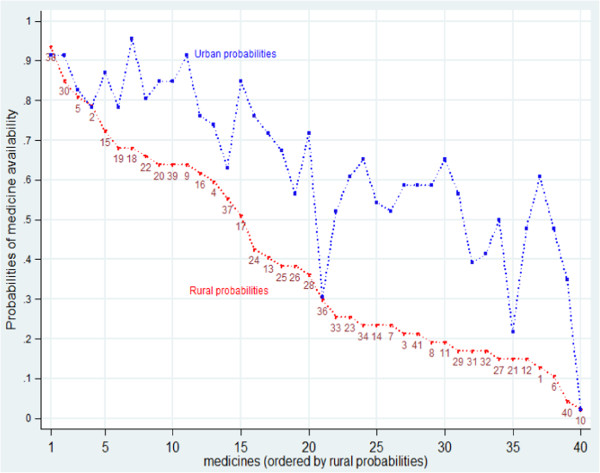
Probability of finding each medicine in rural and urban sample outlets, 2006: medicines ordered by rural probabilities.

Measured by on-the-shelves availability on a given day, in health facilities and shops, access to medicines in Tanzania is systematically worse in rural than urban areas [35,36]^h^. Figure 1 presents the evidence on availability from the 2006 survey, in a graphical format that avoids presentation of averages across medicines. The figure charts the probability, for each tracer medicine, of its availability in a sample facility or shop, in a rural or in an urban area, on the day of the 2006 survey. The data points are thus the proportion of the facilities and shops, in urban and rural areas respectively, that stocked the medicine on the survey day. The medicines are ordered by *rural* availability, allowing ease of comparison with urban availabilities. The numbers identify individual medicines. The web appendix Additional file 1: Table S1 lists the medicines, reference numbers and uses. Some medicines are identified in the text below.

Figure 1 shows that in 2006 urban availability of each tracer medicine was consistently above or in a few cases equal to rural availability. The four medicines most widely available in rural areas (probability > 75%) in 2006 were the then first line anti-malarial treatment (Sulphadoxine with Pyramethamine (SP), 38), an anti-amoeba medicine and an anti-worm treatment (Metronidazole and Albendazole, 30, 2) and a basic antibiotic (Amoxicillin, 5). For these, the urban/rural gap was also small (Figure 1). The eleven other medicines with rural availability >50% included 5 other antibiotics (including a syrup for children and an injectable); two other anti-malarials, an injectable and a syrup; Folic acid for anaemia (22); an anti-inflammatory (Diclofenac, 18); a treatment for bilharzia (Prazequantel, 37) and a tranquillizer (Diazepam, 17).

The urban bias evident right across the sample of tracer medicines in 2006 (Figure 1) persists in the 2009 data (see web appendix Additional file 1: Figure S1), with some narrowing of the extent urban bias. There were few differences between in the medicines most widely available in rural areas in 2006 and 2009. The main shift is a reduction in availability of SP (38) in 2009 in favour of the new first line anti-malarial, Arthemeter and Lumefantrin (Alu) (6).

The average rural availability in 2006 was 40%, while the average urban availability was 64%, a difference of 24 percentage points (Table 1). In 2009, this difference had declined to 18 percentage points (Table 1 row 3). The last row in Table 1 uses the z-test to test for equality between the rural and urban probabilities (the null hypothesis). In both years, the null hypothesis is rejected, that is, the rural and urban probabilities differ significantly^i^.

**Table 1:** Probability of finding a tracer medicine in a sample outlet, by rural and urban location, 2006 and 2009 (40 medicines)

**Location**	**2006**			**2009**		
	**Probability**	**Standard error**	**Sample size**	**Probability**	**Standard error**	**Sample size**
**Rural**	0.40	0.0113	1880	0.42	0.011	1880
**Urban**	0.64	0.0112	1840	0.60	0.012	1800
***Difference***	*-0.24*	*0.0159*		*-0.18*	*0.016*	
**Two sample test of proportions:**	z-test = -14.6			z-test = -11.1		
H_0_ : P_rural_ = P_urban_	Pr(|Z| < |z|) = 0.0000			Pr(|Z| < |z|) = 0.0000		
H_a_ : P_rural_ ≠ P_urban_	*Null-hypothesis is rejected*			*Null-hypothesis is rejected*		

Given the high availability of a few medicines, the low rural average availability reflects a long tail of essential medicines largely unavailable in rural but not in urban areas (Figure 1). The urban bias in medicines availability is a persistent and serious policy concern.

### Absence of urban bias in access to medicines manufactured in Tanzania

In field research on medicines access in rural Tanzania in 2006-7, we were surprised to find that a disproportionate share of medicines actually available in rural shops and in private and faith-based facilities had been made in Tanzania [5] p.456. For seven tracer medicines that were licensed for sale in drug shops in 2006, 66% of the availability was from Tanzanian producers, while in the non-governmental health facilities, paediatric suspensions, basic antibiotics, anti-malarials and analgesics from Tanzanian suppliers were widely stocked.

**Figure 2:**
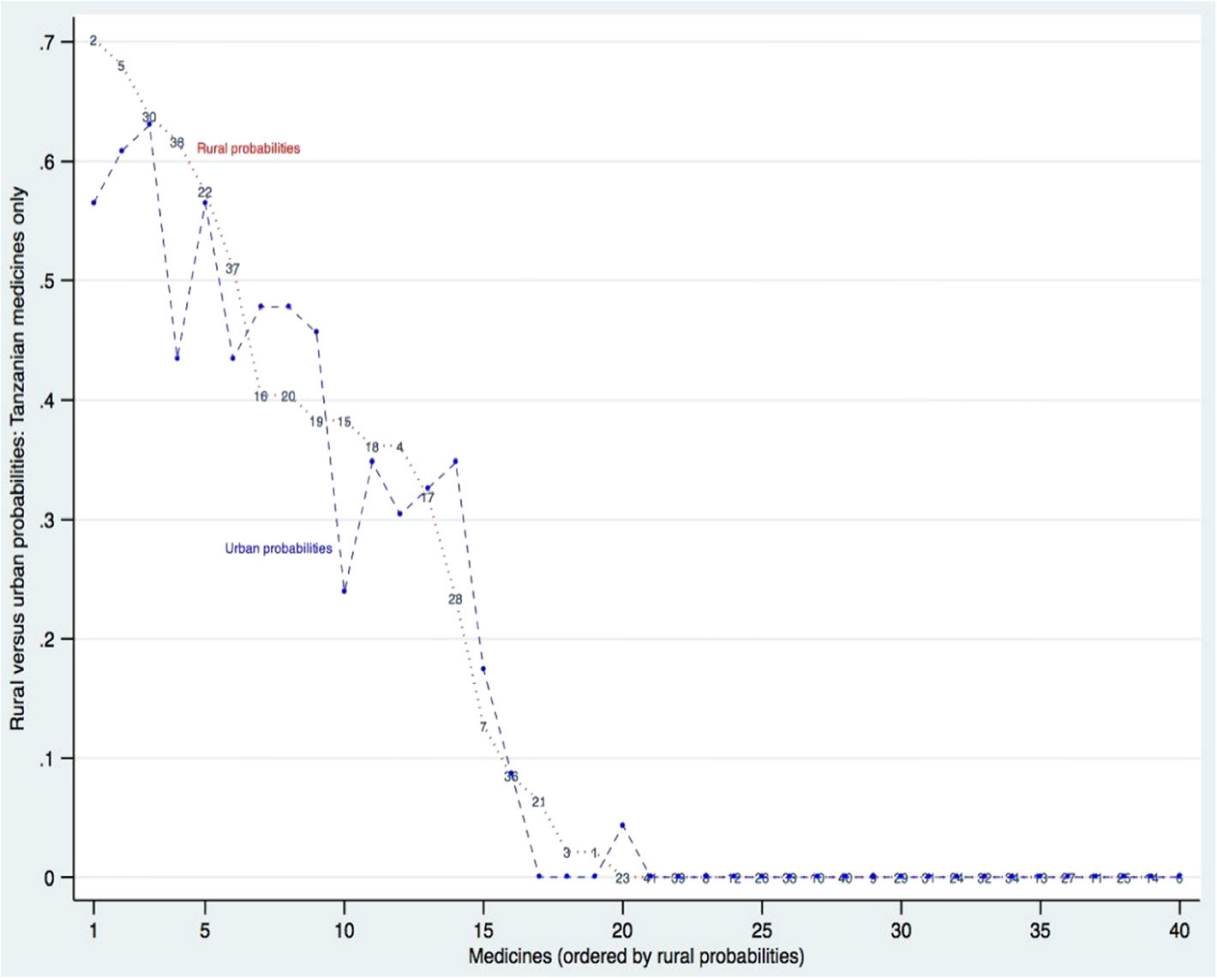
Probability of finding each medicine in rural and in urban sample outlets 2006: medicines manufactured in Tanzania only, ordered by rural probabilities.

This finding led to the exploration of the WHO/HAI data presented here. Figure 2 compares rural and urban availability for each medicine supplied from Tanzanian manufacturers. Half of the 40 tracer medicines in this study were found in the sample outlets in 2006 as products from Tanzanian manufacturers (the number in 2009 was 21). The medicines are ordered in Figure 2 by rural probability, and as Figure 2 shows, the probability of finding a Tanzanian manufactured medicine was approximately equal in rural and in urban outlets. There is no urban bias.

The data from 2009 confirm this finding (web appendix Additional file 1: Figure S2). While the pattern of urban and rural availability is less closely aligned in 2009 there is no over all urban bias in the availability of Tanzanian medicines in that year either, and indeed there is some rural advantage. Note that in 2006, the probability of finding certain Tanzanian medicines was quite high: there were 9 such medicines for which the rural probability in 2006 was >40%. Those probabilities were lower in 2009: the share of Tanzanian manufactures in total medicines availability appears to have declined.

Table 2 confirms that there was no significant difference between the probability of finding a tracer medicine manufactured in Tanzania in a rural and in an urban outlet, in 2006 or in 2009. It also confirms the general drop in availability of Tanzanian medicines in the later year.

**Table 2:** Probability of finding a tracer medicine manufactured in Tanzania in a sample outlet, by rural and urban location, 2006 and 2009 (40 medicines)

**Location**	**2006**			**2009**		
	**Probability**	**Standard error**	**Sample size**	**Probability**	**Standard error**	**Sample size**
**Rural**	0.172	0.0087	1880	0.113	0.0073	1880
**Urban**	0.163	0.0086	1840	0.097	0.0070	1800
***Difference***	*0.009*	*0.0122*		*0.016*	*0.010*	
**Two sample test of proportions:**	z-test = 0.7588			z-test = 1.587		
H_0_ : P_rural_ = P_urban_	Pr(|Z| < |z|) = 0.4480			Pr(|Z| < |z|) = 0.112		
H_a_ : P_rural_ ≠ P_urban_	*Null-hypothesis is accepted*			*Null-hypothesis is accepted*		

### Urban bias in availability of imported medicines

**Figure 3:**
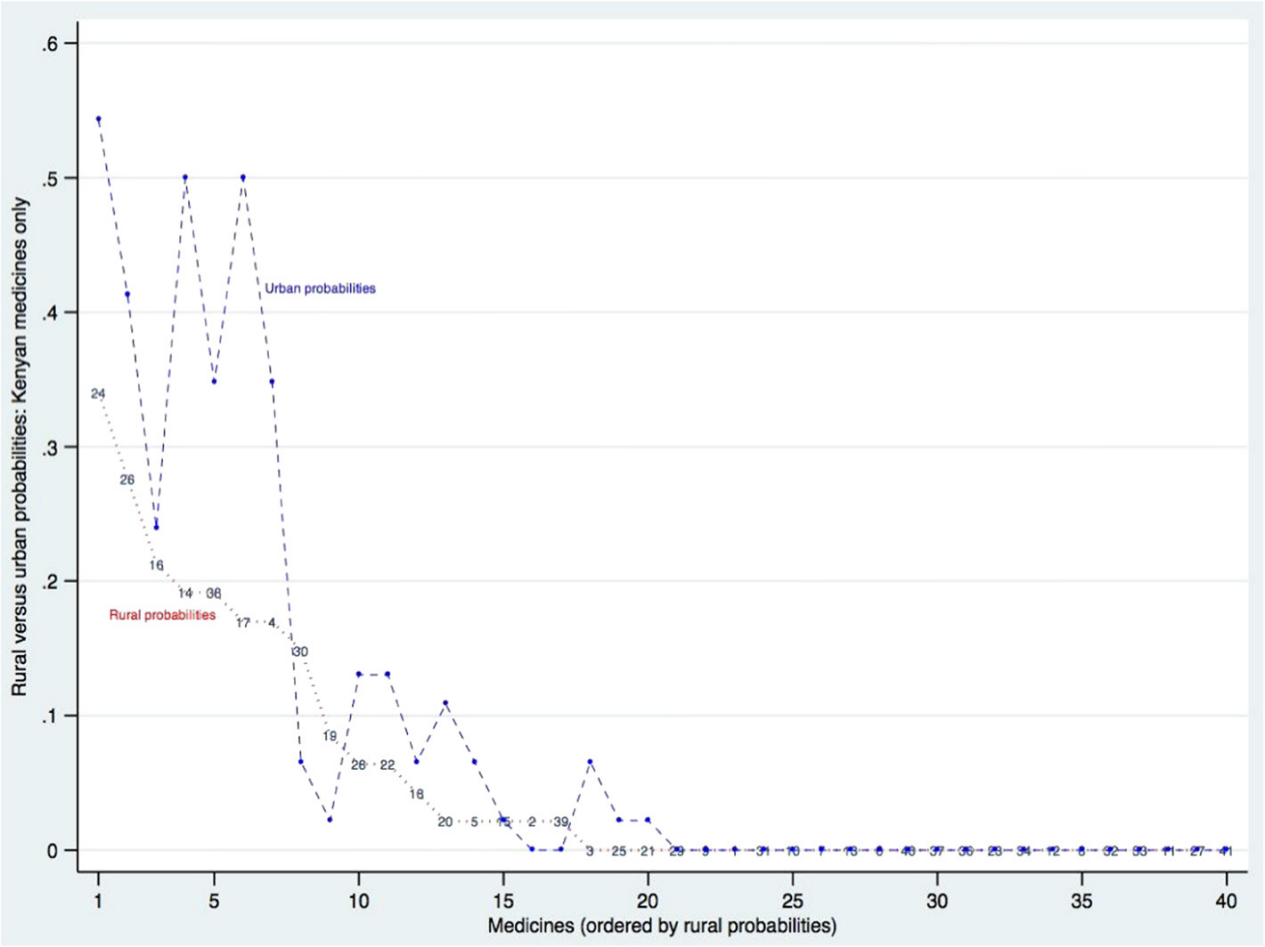
Probability of finding each medicine in rural and in urban sample outlets 2006: medicines manufactured in Kenya only, ordered by rural probabilities.

Most non-Tanzanian medicines available in the sample outlets were imported from outside East Africa. There were 20 medicines for which some supplies were imported from Kenya, but probabilities were generally quite low. Just five had availability >25% in 2006: two anti-malarials (4, 38), a diuretic (24), a tranquilliser (17), and anti-bacterial eye drops (14). Figure 3 compares rural and urban availability for each medicine supplied by Kenyan manufacturers, showing that there is some urban bias in availability of the Kenyan medicines. This urban bias persists in 2009 (see web appendix Additional file 1: Figure S3).

Table 3 confirms this urban bias finding for medicines manufactured in Kenya. In both years, the probability of finding a medicine made in Kenya was significantly lower in rural than in urban areas. The Kenyan medicines were also less available than Tanzanian medicines over all, in both urban and rural areas, in both years (compare Tables 2 and 3, rows 2 and 5).

**Table 3:** Probability of finding a medicine manufactured in Kenya in a sample outlet, by rural and urban location, 2006 and 2009 (40 medicines)

**Location**	**2006**			**2009**		
	**Probability**	**Standard error**	**Sample size**	**Probability**	**Standard error**	**Sample size**
**Rural**	0.052	0.0051	1880	0.048	0.0049	1880
**Urban**	0.090	0.0067	1840	0.083	0.0070	1800
***Difference***	*-0.039*	*0.0084*		*0.034*	*0.0082*	
**Two sample test of proportions:**	z-test = -4.595			z-test = -4.222		
H_0_ : P_rural_ = P_urban_	Pr(|Z| < |z|) = 0.0000			Pr(|Z| < |z|) = 0.0000		
H_a_ : P_rural_ ≠ P_urban_	*Null-hypothesis is rejected*			*Null-hypothesis is rejected*		

**Figure 4:**
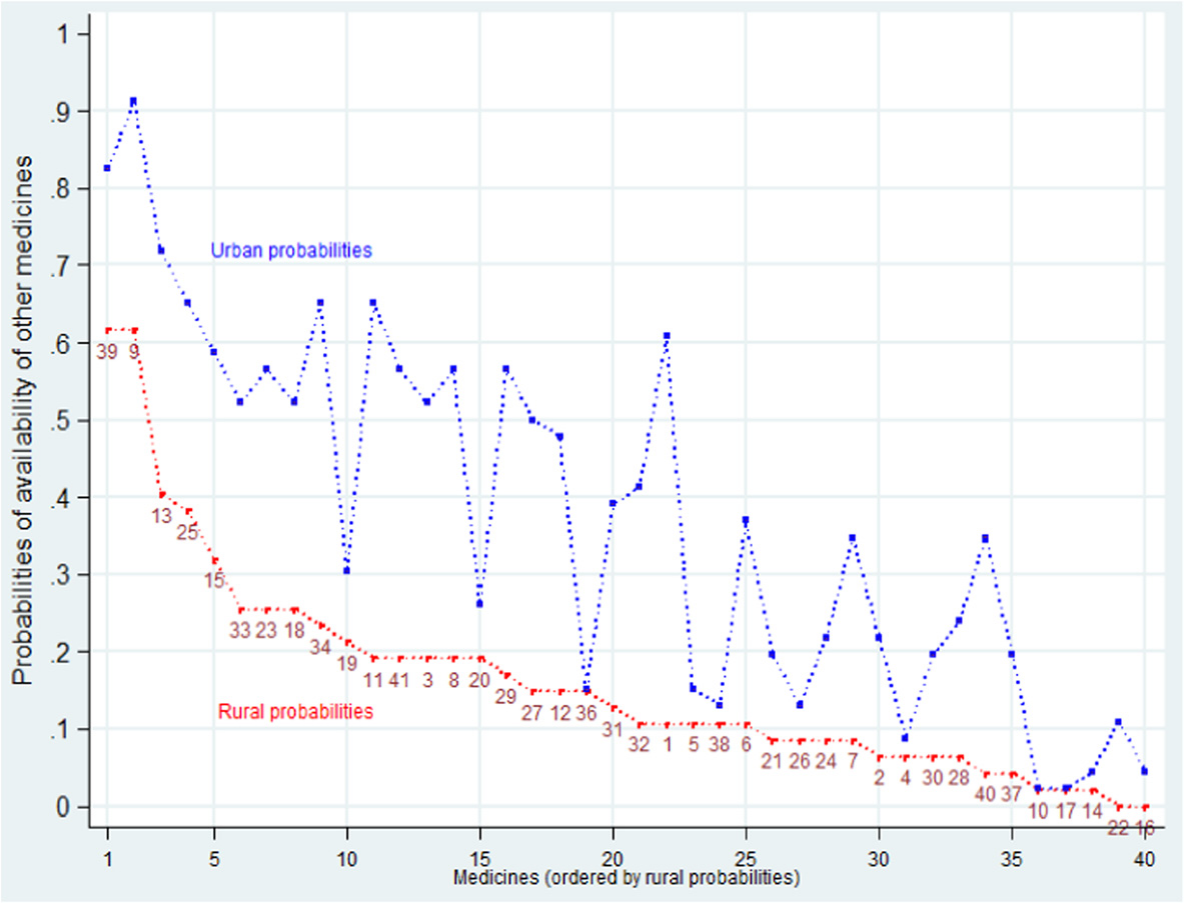
Probability of finding each medicine in rural and in urban sample outlets 2006: medicines manufactured outside Tanzania and Kenya only, ordered by rural probabilities.

The main source of urban bias in medicines availability was however from medicines supplied from manufacturers outside Tanzania and Kenya, here called 'other imports’. These were mainly sourced from Indian manufacturers. 'Other imports’ were found for every tracer medicine in the survey in both years. Figure 4 compares rural and urban availability of other imports of each medicine in 2006. Figure 4 shows consistent urban bias across the whole set of tracer medicines. Unlike Tanzanian-manufactured medicines, other imports were much more likely to be found in urban shops and facilities than in rural outlets. Only three medicines showed equal urban and rural availability, all with *low* availability: an asthma inhaler (10), a tranquilliser (17) and an anti-epilepsy medicine (36).

Additional file 1: Figure S4 in the web appendix shows the equivalent figure for 2009. The urban bias persists across the sample in 2009, though it appears somewhat smaller, and there are two medicines (Folic Acid, for anaemia, important in pregnancy, and Furosemide, a diuretic) that have wider rural than urban availability from other imports in 2009. Table 4 confirms the impression of a highly significant urban bias in both years: the probability of finding a medicine imported from outside Tanzanian and Kenya in a rural area was very significantly lower than the urban probability in both 2006 and 2009, though the bias in 2009 was smaller and rural availability higher (Table 4).

**Table 4:** Probability of finding a medicine manufactured outside Tanzania or Kenya in a sample outlet, by rural and urban location, 2006 and 2009 (40 medicines)

**Location**	**2006**			**2009**		
	**Probability**	**Standard error**	**Sample size**	**Probability**	**Standard error**	**Sample size**
**Rural**	0.163	0.0085	1880	0.251	0.0100	1880
**Urban**	0.375	0.0113	1840	0.412	0.0116	1800
***Difference***	*-0.212*	*0.014*		*-0.160*	*0.0160*	
**Two sample test of proportions:**	z-test = -14.575			z-test = -10.324		
H_0_ : P_rural_ = P_urban_	Pr(|Z| < |z|) = 0.0000			Pr(|Z| < |z|) = 0.0000		
H_a_ : P_rural_ ≠ P_urban_	*Null-hypothesis is rejected*			*Null-hypothesis is rejected*		

In summary, the entire rural disadvantage in access to medicines in Tanzania, relative to the urban access, is shown in these data sets to be attributable to urban bias in the distribution of imported medicines – and notably in non-Kenyan imports that come largely from the Indian sub-continent. The graphical analysis shows that this is the case right across the sample of medicines: the average bias is not generated by a few outliers. The urban bias in access to imports is mitigated only by equitable rural/urban distribution of Tanzanian-made medicines.

Finally, we can compare the rural and urban availabilities of two categories of these medicines: those available as *both* Tanzanian-manufactured and imported items, and those available *only* as imports. Table 5 shows that in both years, the rural and the urban availability of these essential medicines was higher for medicines that were both manufactured in Tanzania and imported as compared to the subset of medicines available only as imports. The rural disadvantage in availability of these medicines was particularly severe in relation to medicines available only from imports in both years (Table 5). It follows that more local production, both of those medicines already locally produced, and also of medicines currently available solely as imports, may improve rural availabilities.

**Table 5:** Probability of finding a tracer medicine in a sample outlet, by rural and urban location and manufacturing origin, 2006 and 2009 (40 medicines)

**Location**	**2006**		**2009**	
	**Manufactured in Tanzania and imported (20 medicines)**	**Imported only: not manufactured in Tanzania (20 medicines)**	**Manufactured in Tanzania and imported (21 medicines)**	**Imported only: not manufactured in Tanzania (19 medicines)**
**Rural**	0.53 *(0.016)*	0.26 *(0.014)*	0.49 *(0.016)*	0.34 *(0.016)*
**Urban**	0.71 *(0.015)*	0.56 *(0.016)*	0.63 *(0.016)*	0.58 *(0.017)*
***Difference***	-0.18 *(0.022)*	-0.30 *(0.022)*	-0.14 (0.023)	-0.24 (0.023)
**Two sample test of proportions:**	z-test = -7.91	z-test = -13.27	z-test = -5.93	z-test = -9.91
H_0_ : P_rural_ = P_urban_	Pr(|Z| < |z|) = 0.0000	Pr(|Z| < |z|) = 0.0000	Pr(|Z| < |z|) = 0.0000	Pr(|Z| < |z|) = 0.0000
H_a_ : P_rural_ ≠ P_urban_	*Null-hypothesis is rejected*	*Null-hypothesis is rejected*	*Null-hypothesis is rejected*	*Null-hypothesis is rejected*

Note: sampling errors in brackets (in *italics*).

## Discussion: explaining urban bias

What factors may explain the observed urban bias, and to what extent can supporting evidence be found in these data or the relevant literature to support these explanations?

A first hypothesis may be that the urban bias is a feature of private market distribution, while public sector distribution of imported medicines is bias-free^j^. The public distribution system serves mainly the public facilities, and the public facilities source almost all medicines from the public distributor, the Medical Stores Department (MSD) [33]. Therefore this hypothesis can be tested using these data by examining the extent to which the availability of medicines in public facilities, sourced from Tanzanian manufacturers and from imports, displays urban bias.

As Table 6 shows, the hypothesis is rejected. The urban bias in availability of other imports of medicines is highly significant in the public sector, as in the whole sample in 2006. Additional file 1: Table S2 in the web appendix shows that this was also the case in 2009. The unbiased distribution of medicines made in Tanzania is confirmed for the public sector sub-sample. The probability of finding a Kenyan medicine in the public sector outlets is very low in both years, while the urban bias of the other imports is highly significant in the public sector sub-sample alone.

**Table 6:** Probability of finding a tracer medicine in a public sector sample outlet, by rural and urban location and manufacturing origin, 2006 (40 medicines)

**Location**	**All medicines**		**Tanzanian medicines**		**Kenyan medicines**		**Other medicines**	
Rural	0.34	*(0.011)*	0.206	*(0.016)*	0.005	*(0.003)*	0.12	*(0.013)*
*Sample size = 640*								
Urban								
Sample size = 600	0.59	*(0.011)*	0.252	*(0.017)*	0.038	*(0.008)*	0.29	*(0.018)*
Difference	-0.25	*(0.0159)*	-0.045	*(0.024)*	-0.034	*(0.008)*	-0.17	*(0.022)*
z-test	-8.9		*-1.90*		-4.13		-7.53	
H_0_: P_rural_ = P_urban_	Pr(|Z| < |z|) = 0.0000		Pr(|Z| < |z|) = 0.057		Pr(|Z| < |z|) = 0.000		Pr(|Z| < |z|) = 0.0000	
H_a_ : P_rural_ ≠ P_urban_	*Null hypothesis is rejected*		*Null hypothesis is accepted*		*Null hypothesis is rejected*		*Null hypothesis is rejected*	

Note: sampling error in brackets (in *italics*).

A second hypothesis is that, given the reliance on out-of-pocket spending for access to medicines in all sectors in Tanzania, and the extensive reliance of the population on purchase of medicines from shops [33], the urban bias among imported medicines may result from the lower purchasing power found in the rural areas. That is, outlets in rural areas, including public sector and faith-based facilities, may stock only those medicines that rural consumers can afford. If the imported medicines are predominantly those in the higher price bracket, then that might explain the observed tendencies for the imported medicines to 'stick’ in urban areas. Conversely, if the medicines produced in Tanzania are in the lower price bracket, then that may explain their more even rural/urban distribution.

**Figure 5:**
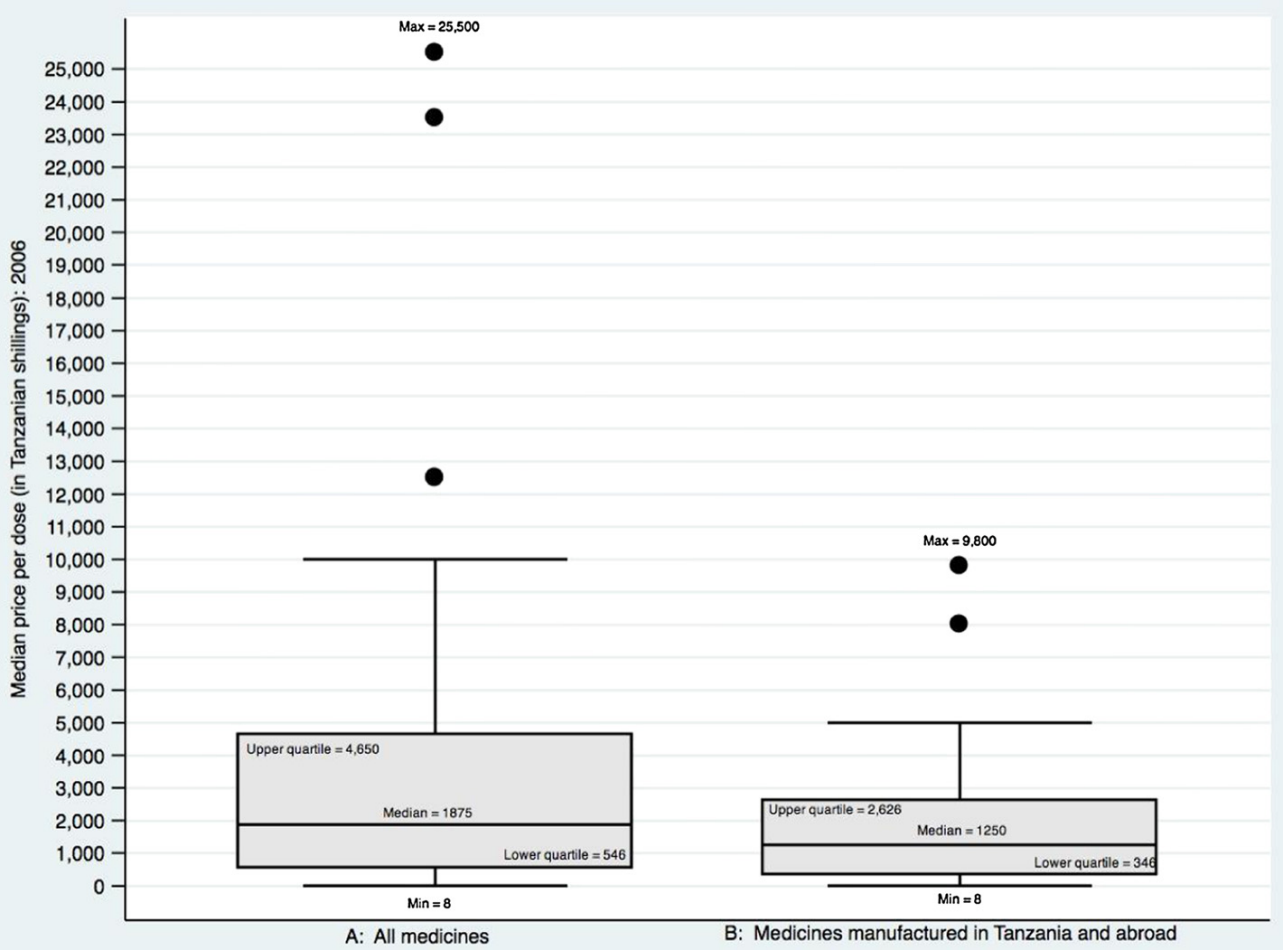
Median price per dose per medicine 2006: all tracer medicines (panel A) compared to the subset of those medicines both produced in Tanzania and also imported (panel B).

Figure 5 shows that in 2006 the medicines manufactured in Tanzania did tend to be in lower price brackets as compared to the set of all the tracer medicines. The figure also shows however that some medicines manufactured in Tanzania were in the upper quartile of prices in the sample. Furthermore, the price range of medicines produced in Tanzania was significantly higher in relation to the sample as a whole in 2009 (see web appendix Additional file 1: Figure S5). Tanzanian producers sold a range of medicines, including some of the higher priced items, and the lack of urban bias is consistent across all items.

**Figure 6:**
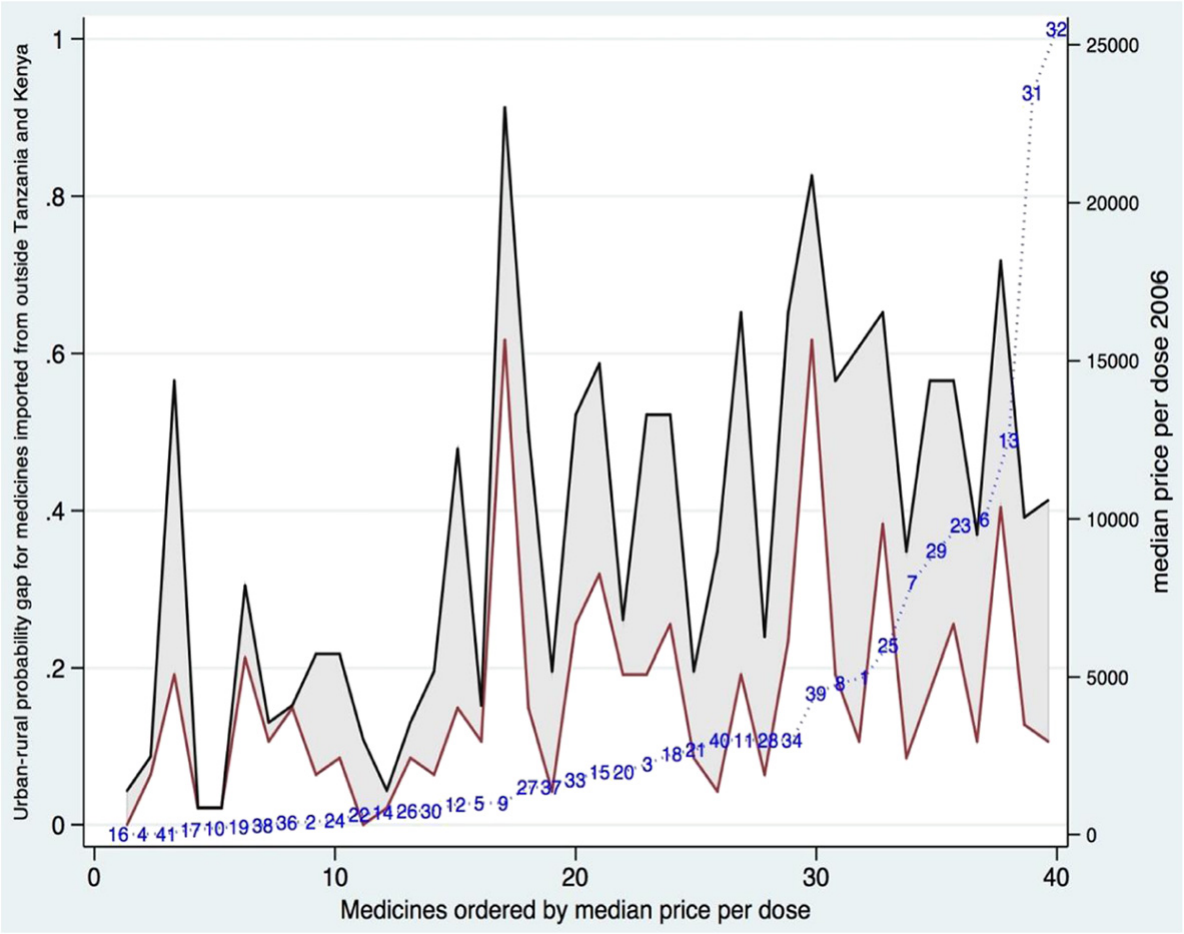
Urban-rural probability gap 2006: medicines imported from outside Tanzania and Kenya, ordered by median price per dose of each medicine.

If it were the case that the more expensive imported medicines had a larger urban bias than the less expensive items, this would provide partial support for the view that urban bias in imports is influenced by low rural purchasing power. Figure 6 is designed to address this hypothesis. It shows the rural/urban probability gap for other imports. The medicines are ordered by price per dose (right hand axis). The lower line on the graph shows the rural availability of each medicine; the higher line is the urban availability. The distance between the two lines – highlighted as a shaded area – shows the rural/urban probability gap in availability of other imports of each medicine.

As Figure 6 shows, the urban bias in access to other imports runs right across the price spectrum, though the larger probability gaps do tend to be at the higher-priced end. There are just six medicines at the lower price end showing no or little urban bias, and all with low urban and rural import availability. Of these, three had high (over 50%) *general* rural availability driven by local and Kenyan production (the then first line anti-malarial, SP (38), a paediatric antimalarial (4), and a tranquilliser (17), see Figures 1 and 2). The other three medicines (antibiotic eye drops (14), and anti-epileptic (36) and an asthma inhaler (10)) were other-imports-only items, with general low availability across the whole sample despite a low price per dose.

The equivalent Additional file 1: Figure S6 for 2009 in the web appendix shows a similar picture. The urban bias is slightly lower over all, and it again tends to be higher for the higher priced medicines, while the availability of lower priced imported items tends to be quite low in rural *and* urban areas. It appears therefore to be the case that higher priced imports are somewhat more subject to urban bias than cheaper imported medicines, perhaps because of lower rural purchasing power. But this cannot offer a complete explanation of the urban bias in imports, since some lower priced items also display bias (Figure 6).

What other likely explanatory factors can be identified from the literature? Part of the explanation may lie in the organisation of distribution channels for local manufactures and for imports. Global medicines supply chains are complex and risky, with problematic distribution of risks [37], while local manufacturers such as Shelys, the largest pharmaceutical producer in Tanzania, have shorter supply chains when selling into the home market. For Tanzania-based manufacturers, the home market is generally their core business, and potentially they have greater market information, and flexibility to respond to market needs. One manufacturer in Tanzania explained^k^ that the firm operated its own distribution network to ensure wide distribution. He also noted that medicine packets include information in Kiswahili, increasing accessibility and trust for purchasers, small shop keepers and lower level professional staff. Some of the locally produced items – for example, branded generics from Shelys such as Sheladon paracetamol – had brand recognition in shops^l^.

There is also some evidence that rural Tanzanian consumers have developed trust in Tanzanian medicines as compared in Indian competitors. Exit interviews with medicines purchasers in 69 private and NGO rural medicines outlets (shops and facilities) were asked in 2006 about their preferences for country of origin of medicines for three example illnesses; of those who expressed an opinion, more than half preferred Tanzanian medicines over imports for pneumonia and diarrhoea, while over a third preferred Tanzanian medicines for malaria^m^. European medicines came next in terms of preferences, with Kenyan and Indian medicines trailing well behind.

Conversely many private wholesalers represent individual Indian manufacturers, and are known to concentrate on urban distribution to facilities and pharmacies. Interviewees in 2010^n^ confirmed that few Dar es Salaam-based wholesaler/importers had up-country distribution networks. Urban consumers are also more likely to demand imported medicines – in particular, high-priced European brands – and the importers tend to concentrate on supplying urban markets [5].

Interviews and recent studies of distribution of subsidised imported anti-malarials in Tanzania confirm that for importers to distribute widely up-country required the building of much improved distribution networks. From 2006 Tanzania switched from its previous first line anti-malarial medicine, Sulphadoxine-Pyrimethamine (SP), to fixed combination Artemisinin-based medicines (ACTs). Two pilot experiences of distribution of subsidised imported ACTs to private shops were undertaken.

One pilot, supported by Management Sciences for Health (MSH), and US-funded^o^, distributed the medicines to licensed drug shops called Accredited Drug Dispensing Outlets (ADDOs). A wholesaler interviewed in 2010^p^ described the large amount of travelling and persuasion, and adaptation of delivery networks, required to encourage uptake by rural shopkeepers. Studies of the second pilot distribution of ACTS to shops in two districts, supported by the Clinton Foundation, from 2007 [38,39], found that the distribution of both ACTS and locally produced SP was patterned by geography, remoteness being associated with lower rates of stocking; however the Kenyan version of SP was very widely available in the studied districts, with no difference in stocking by remoteness. The study notes that the Kenyan medicine was somewhat cheaper than the Tanzanian competitor, and that the differences may also be due to different distribution patterns, but no evidence was available on the latter issue^q^.

While the organisation of private distribution networks may provide part of the explanation for private sector urban bias in distribution of imported medicines, the finding of a similar bias in public sector distribution remains a puzzle. The Medical Stores Department (MSD), the public sector medicines distributor, should in principle be supplying equally to rural and urban facilities, yet imported medicines were more likely to be found in the urban than in the rural public sector.

A partial explanation for this finding may lie in the urban location of large hospitals in Tanzania, since these are the facilities likely to be using the more complex medicines that are also likely to be sourced only from imports. However this does not offer a complete explanation, since urban bias in public sector availability of imported medicines is observed for most medicines in this data set, whether import-only or available from both local manufacturers and importers. Another explanatory factor may be that urban facilities are more likely than rural facilities to raise and retain fees which can be used for medicines purchases to fill gaps in supply. The sources of public sector urban bias, including the balance between distribution decisions by the Medical Stores Department and the outcomes of individual health facility procurement decisions, deserve further investigation^r^.

Finally, a comparison of the 2006 findings, discussed in detail here, with the 2009 findings in the web appendix shows that the share of Tanzanian-based pharmaceutical manufacturers in their local market appears to be falling. This is a cause for concern, because this article’s findings suggest that this trend may increase the relative disadvantage of the rural population in access to essential medicines. It will also, of course, reduce industrial employment and development within the country. The decline may be in part the result of the switch from local production to imports of first line anti-malarials, previously a major product for several Tanzanian manufacturers, but this is unlikely to be the full explanation.

## Conclusion

This article has employed mainly graphical analysis to demonstrate that the medicines surveys for Tanzania for 2006 and 2009 identify a pattern of urban bias in access to imported medicines, while confirming that access to medicines produced in Tanzania does not display urban bias. The article has also shown that urban bias in imported medicines generally exists across the full range of the 40 tracer medicines in the WHO/HAI studies from which the data are drawn.

The article thus provides *prima facie* evidence that locally produced medicines are more accessible than imports for rural consumers, and that medicines which are both imported and locally produced display greater rural and urban availability than those which are import-only. Since the most severe poverty in Tanzania is in rural areas, and the rural population have persistently lower access to medicines in general, this evidence suggests that building up local production may support improved rural access to medicines. The local production of medicines appears to be an area of policy with potential synergies between industrial and health policy objectives.

As the discussion noted, these empirical findings are new, and they remain largely unexplained in the broader literature. This article’s findings indicate that further investigation is required of the reasons for poor rural access to medicines, including the different organisation and performance of distribution channels used by local producers and by importers, and hence of the better performance of local producers in reaching rural consumers identified here.

## Endnotes

^a^Source: interviews forming part of unpublished research in Tanzania by Mackintosh and Mujinja for UNITAID 2010.

^b^See http://www.haiweb.org/medicineprices/ for methods, survey reports and publications from this cross-country data collection exercise.

^c^Oral rehydration salts (ORS) were included in the 2009 tracer medicines list but not in the 2006 survey; to allow systematic comparison of the two years, we have dropped ORS from the data as presented, leaving 40 tracer medicines. The Additional file 1: Table S1 in the web appendix lists the tracer medicines and their uses.

^d^A fourth region was included in 2009; this was dropped from analysis of the 2009 data to allow systematic comparison of the two years.

^e^'Non-governmental Action to Improve the Access of the Poor to Good Quality Low Cost Drugs’ 2006-8, funded by the UK ESRC. See acknowledgements. The full research report (2009), and project publications, are available at http://www.esrc.ac.uk/my-esrc/grants/RES-155-25-0046/read

^f^Unpublished report to UNITAID by Mackintosh and Mujinja with Justin-Temu, entitled 'Interactions between Global Policy and Local Markets and Production of Medicines: a case study in Tanzania’ 2011.

^g^A search for “urban bias” in Pubmed produced 56 references, including [40] on NGOs and [41] among many on health workers; also [42] on HIV treatment; searches did not find other assessments of the type in this paper on medicines distribution.

^h^The WHO/HAI datasets do not appear to have been systematically analysed for rural vs. urban disadvantage: in cross-country and summary publications; this is not a theme discussed, for example, in [12] or [43,44]; individual country reports such as [35,36] however do report this divergence.

^i^The last row in Table 1, and in the subsequent tables, shows the results of the tests for the difference between two proportions under the null-hypothesis that the difference is zero. The relevant test statistic is the z-test, based on the normal distribution. The results in Table 1 show the computed value of the z-test statistic and its probability value.

^j^This hypothesis was suggested by a Tanzanian official at a presentation of an early draft of these findings.

^k^Interview by Mujinja and Mackintosh 2010.

^l^Unpublished data from the study cited in footnote e.

^m^Unpublished data from the study cited in footnote e.

^n^Interview by Mujinja and Mackintosh 2010.

^o^See http://www.msh.org/news-events/news/accredited-drug-dispensing-outlets-in-tanzania-an-example-of-successful-private

^p^Interview by Mujinja and Mackintosh 2010.

^q^See also [45].

^r^A referee suggested that different patterns of burden of disease in urban and rural areas [46] might provide a further explanation of this finding. These disease burden differences have been narrowing in Tanzania [46]. However the fact that our urban bias finding holds across our whole data set suggests these differences may not be a key explanatory variable.

## Competing interests

The authors declare that they have no competing interests.

## Authors’ contributions

MJT led the data collection of the WHO/HAI Tanzanian data that form the basis for this article, and supervised data entry and coding. PM led the data collection for the 2007 and 2010 fieldwork cited, and with MM undertook the analysis that produced the idea for this paper. MW undertook the data analysis of the WHO/HAI data. MM with PM led the drafting of the article. All authors contributed to discussion of data analysis, results and policy conclusions; debated successive drafts; and agreed the final version of the article. All authors read and approved the final manuscript.

## Supplementary Material

Additional file 1 Web appendix tables and figures. Click here for file
