# Supplementary material for: Local production of pharmaceuticals in Africa and access to essential medicines: 'urban bias’ in access to imported medicines in Tanzania and its policy implications
Source: Global Health. 2014 Mar 10;10:12. doi: 10.1186/1744-8603-10-12 (PMC4021829; doi:10.1186/1744-8603-10-12)
Supplement: Additional file 1 — Web appendix tables and figures. [file 1744-8603-10-12-S1.docx]

**Additional File 1: Web Appendix Tables and Figures**

**Table S1 List of tracer medicines with reference numbers and medicine use**

| **Medicine** | **Medicine code** | **Medicine use** |
| --- | --- | --- |
| Aciclovir tab. 200 mg | 1 | anti-viral |
| Albendazole tab. 200mg | 2 | anti-helmentics |
| Amitriptyline tab. 25 mg | 3 | anti-depressant/anti-psychosis |
| Amodiaquine paediatric syrup | 4 | anti-malarial |
| Amoxicillin caps./tab. 250 mg | 5 | anti-bacterial |
| Arthemether +Lumefantrine tab. 20+120mg | 6 | anti-malaria |
| Artesunate tab.100 mg | 7 | anti-malarial |
| Atenolol tab. 50 mg | 8 | anti-hypertensive |
| Benzyl penicillin 5mega units | 9 | anti-bacterial |
| Beclometasone inhaler 50 mcg/ dose | 10 | anti-asthmatic |
| Captopril tab. 25 mg | 11 | anti-hypertensive |
| Carbamazepine tab. 200 mg | 12 | anti-epileptic & anti-convulsant |
| Ceftriaxone inj. 1 g powder | 13 | anti-bacterial |
| Chloramphenical 0.5 % eye drops | 14 | anti-bacterial |
| Ciprofloxacin tab. 500 mg | 15 | anti-bacterial |
| Co-trimoxazole paed. susp. (8+40) mg/mL | 16 | anti-bacterial |
| Diazepam tab. 5 mg | 17 | anti-xiolytic |
| Diclofenac tab. 50mg | 18 | non-steroidal anti-inflammatory |
| Doxycycline cap. 100mg | 19 | anti-bacterial |
| Erythromycin tab. 250 mg | 20 | anti-bacterial |
| Ferrous sulphate tab. 200 mg | 21 | anti-anaemia |
| Folic acid tab.5 mg | 22 | anti-anaemia |
| Fluconazole cap. / tab. 150mg | 23 | anti-fungus |
| Furosemide tab. 40mg | 24 | diuretic |
| Gentamycin inj. 80mg/ml | 25 | anti-bacterial |
| Gentamycin eye/ear drops 1% | 26 | anti-bacterial |
| Glibenclamide tab. 5 mg | 27 | anti-diabetic |
| Griseofulvin tab. 500mg | 28 | anti-fungus |
| Metformin tab. 500 mg | 29 | anti-diabetic |
| Metronidazole tab. 250mg | 30 | anti-amoeba |
| Niverapine/Lamivudine/Stavudine 30 | 31 | anti-viral |
| Niverapine/Lamivudine/Stavudine 40 | 32 | anti-viral |
| Nifedipine retard 20mg | 33 | anti-hypertensive |
| Omeprazole caps. 20 mg | 34 | anti-acid/anti-ulcer |
| Phenytoin 100 mg | 36 | anti-epileptic/anti-convulsant |
| Prazequantel tab. 600 mg | 37 | anti-schistosomiasis |
| Pyrimethamine with sulfadoxine 25+500 mg | 38 | anti-malarial |
| Quinine inj. 300mg/ml | 39 | anti-malaria |
| Ranitidine tab. 150 mg | 40 | anti-ulcer |
| Sulbutamol inhaler 0.1 mg 100 mcg/dose | 41 | anti-asthmatic |

**Table S2 Probability of finding a tracer medicine in a public sector sample outlet, by rural and urban location and by origin, 2009 (40 medicines)**

| **Location** | **All medicines** | | **Tanzanian medicines** | | **Kenyan medicines** | | **Other medicines** | |
| --- | --- | --- | --- | --- | --- | --- | --- | --- |
| Rural  *Sample size = 640* | 0.36 | *(0.019)* | 0.144 | *(0.014)* | 0.022 | *(0.006)* | 0.19 | *(0.015)* |
| Urban  Sample size = 600 | 0.56 | *(0.020)* | 0.143 | *(0.014)* | 0.028 | *(0.007)* | 0.37 | *(0.020)* |
| Difference | -0.25 | *(0.028)* | 0.045 | *(0.020)* | -0.006 | *(0.009)* | -0.18 | *(0.025)* |
| z-test  H_0_: P_rural_ = P_urban_  H_a_ : P_rural_ ≠ P_urban_ | **-6.97**  Pr(\|Z\| < \|z\|) = 0.0000  *Null hypothesis is rejected* | | ***0.02***  Pr(\|Z\| < \|z\|) = 0.98  *Null hypothesis is accepted* | | **-0,73**  Pr(\|Z\| < \|z\|) = 0.47  *Null hypothesis is accepted* | | **-7.08**  Pr(\|Z\| < \|z\|) = 0.0000  *Null hypothesis is rejected* | |

Note: sampling error in brackets (in *italics)*

**Figure S1: Probability of finding each medicine in rural and urban sample outlets, 2009: medicines ordered by rural probabilities**

**
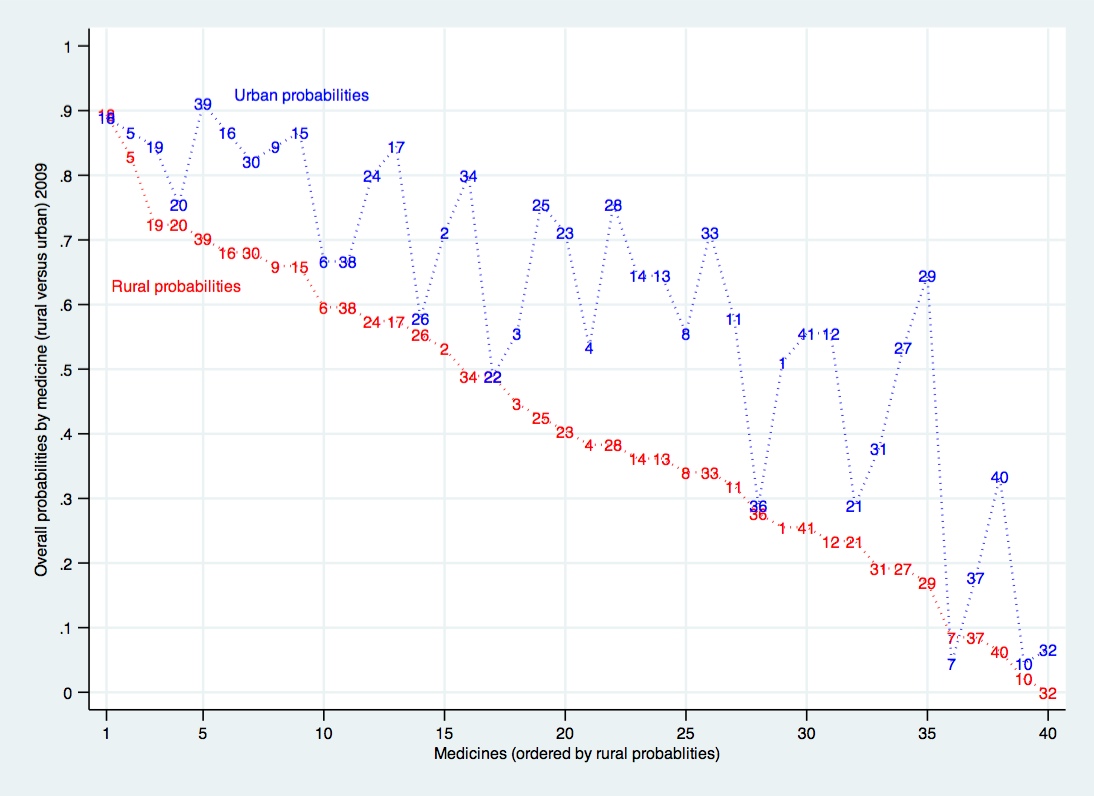
**

**Figure S2: Probability of finding each medicine in rural and in urban sample outlets 2009: medicines manufactured in Tanzania only, ordered by rural probabilities**

**
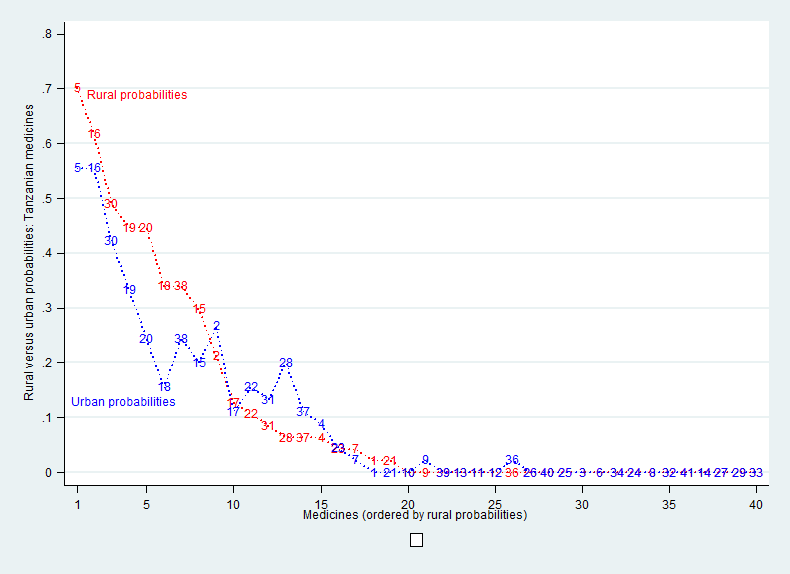
**

**Figure S3: Probability of finding each medicine in rural and in urban sample outlets 2009: medicines manufactured in Kenya only, ordered by rural probabilities.**


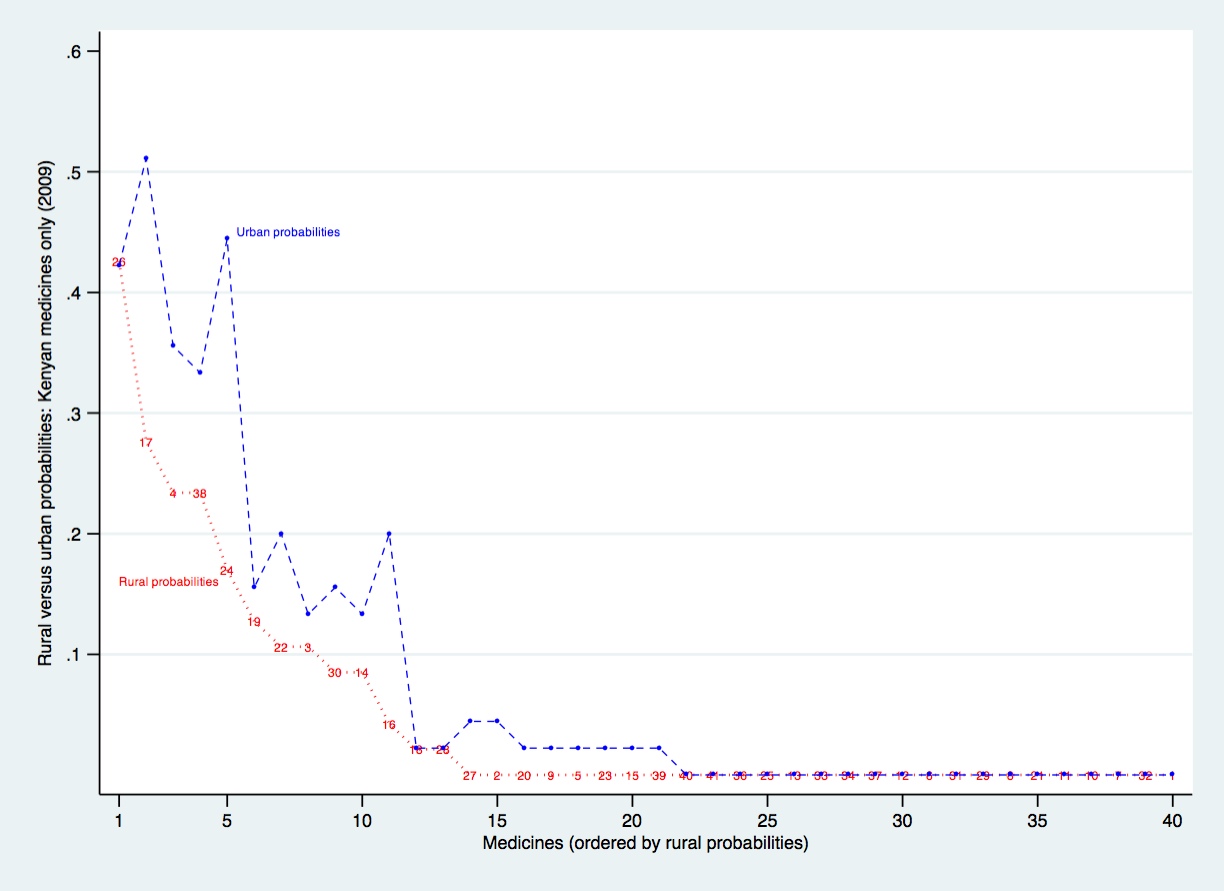


**Figure S4: Probability of finding each medicine in rural and in urban sample outlets 2009: medicines manufactured outside Tanzania and Kenya only, ordered by rural probabilities
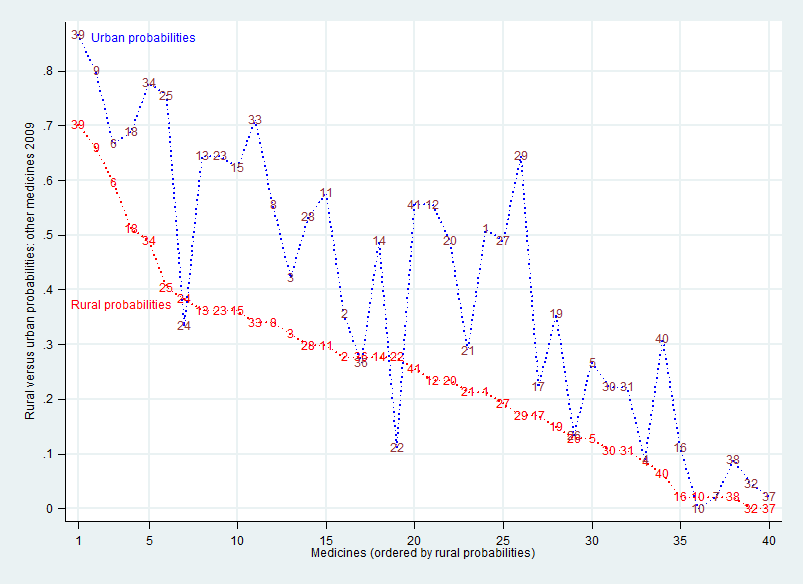
**

**Figure S5: Median price per dose per medicine 2009: all tracer medicines (panel A) compared to the subset of those medicines both produced in Tanzania and also imported (panel B).**

**
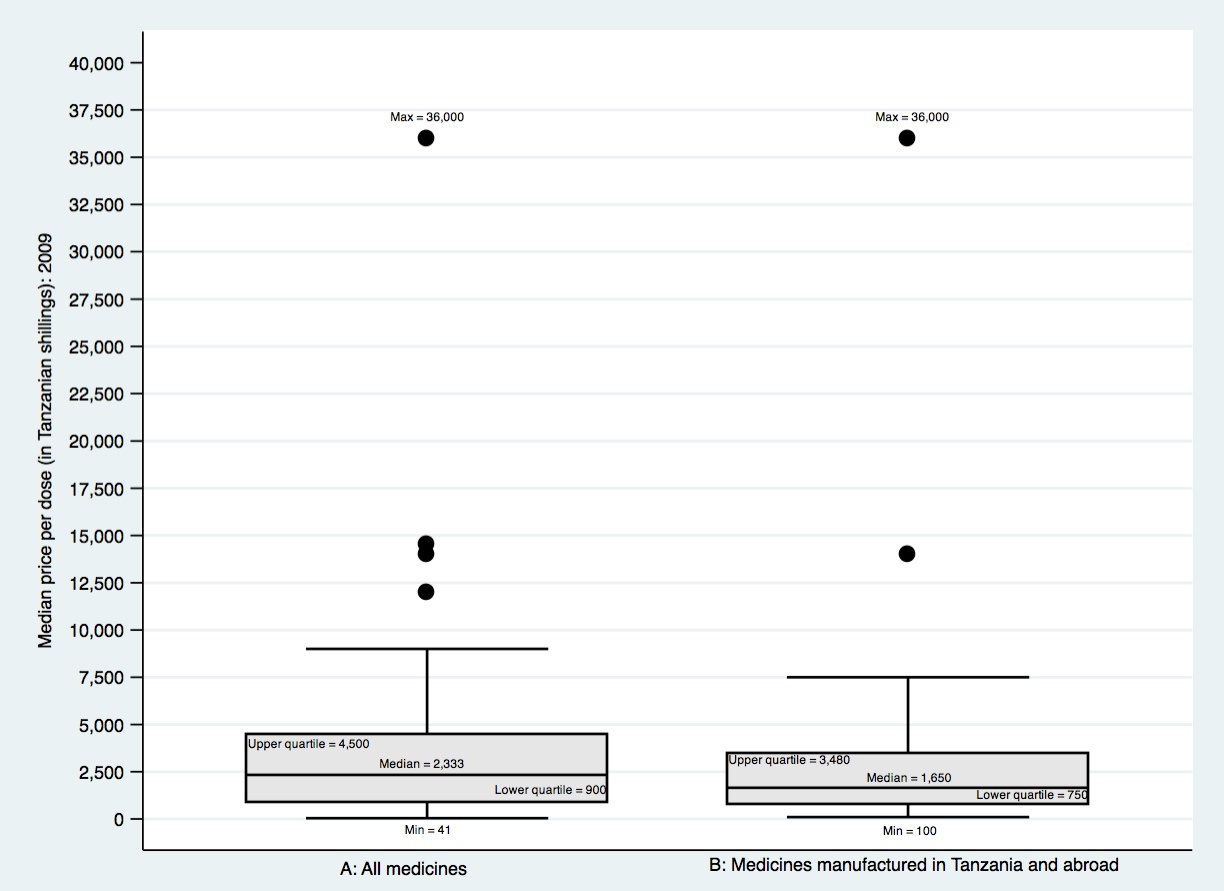
**

**Figure S6: Urban-rural probability gap 2009: medicines imported from outside Tanzania and Kenya, ordered by median price per dose of each medicine**

**
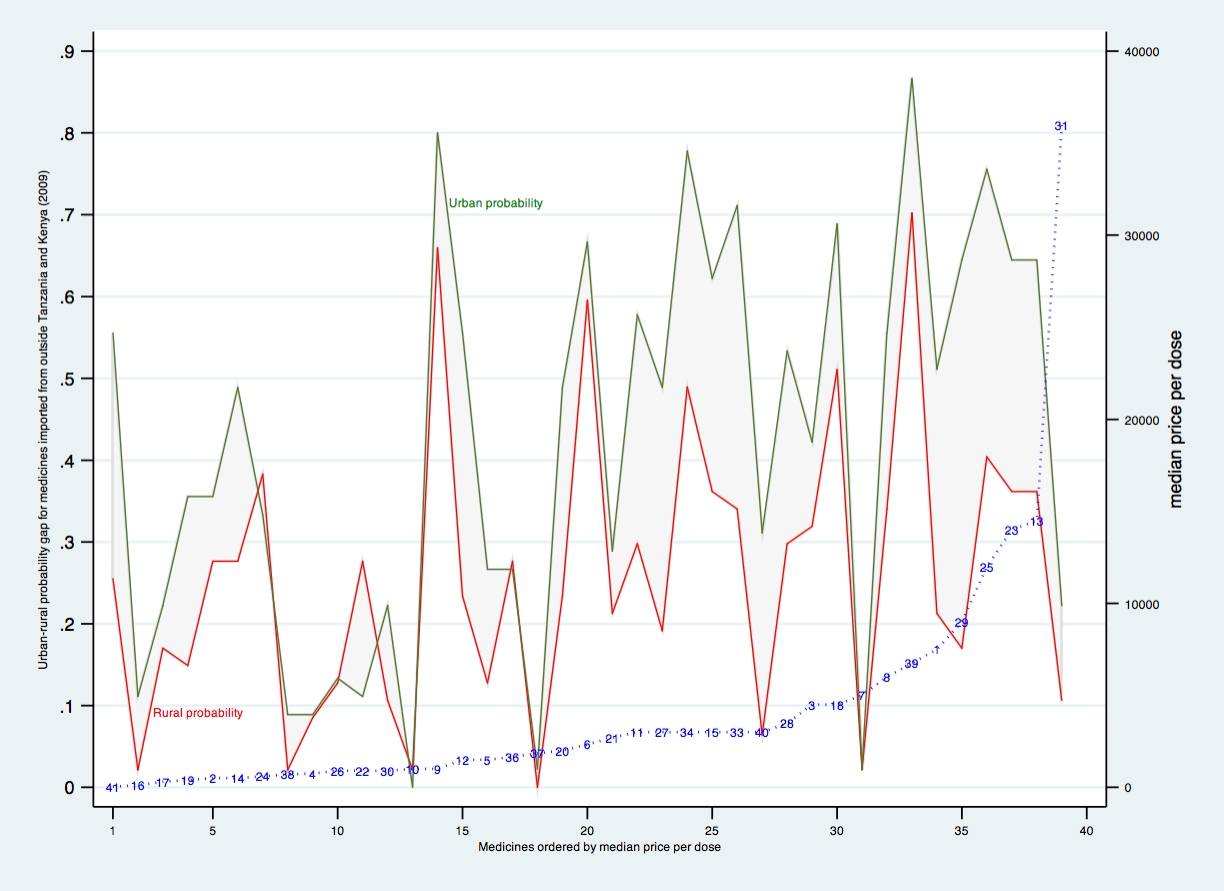
**
